# Characterization of heterogeneous metabolism in hepatocellular carcinoma identifies new therapeutic target and treatment strategy

**DOI:** 10.3389/fimmu.2023.1076587

**Published:** 2023-03-16

**Authors:** Jiabin Yang, Liangtang Zeng, Ruiwan Chen, Shangyou Zheng, Yu Zhou, Rufu Chen

**Affiliations:** ^1^ School of Medicine, South China University of Technology, Guangzhou, Guangdong, China; ^2^ Department of Pancreatic Surgery, Guangdong Provincial People's Hospital (Guangdong Academy of Medical Sciences), Southern Medical University, Guangzhou, China; ^3^ Department of Radiation Oncology, The First Affiliated Hospital, Sun Yat-sen University, Guangzhou, Guangdong, China

**Keywords:** hepatocellular carcinoma, metabolism, differential gene expression, personalized treatment, metabolic subtype, PPT1

## Abstract

**Background:**

Metabolic reprogramming is a well-known hallmark of cancer. Systematical identification of clinically relevant metabolic subtypes of Hepatocellular carcinoma (HCC) is critical to understand tumor heterogeneity and develop efficient treatment strategies.

**Methods:**

We performed an integrative analysis of genomic, transcriptomic, and clinical data from an HCC patient cohort in The Cancer Genome Atlas (TCGA).

**Results:**

Four metabolic subtypes were defined: mHCC1, mHHC2, mHCC3, and mHCC4. These subtypes had distinct differences in mutations profiles, activities of metabolic pathways, prognostic metabolism genes, and immune features. The mHCC1 was associated with poorest outcome and was characterized by extensive metabolic alterations, abundant immune infiltration, and increased expression of immunosuppressive checkpoints. The mHHC2 displayed lowest metabolic alteration level and was associated with most significant improvement in overall survival in response to high CD8+ T cell infiltration. The mHHC3 was a “cold-tumor” with low immune infiltration and few metabolic alterations. The mHCC4 presented a medium degree of metabolic alteration and high CTNNB1 mutation rate. Based on our HCC classification and in vitro study, we identified palmitoyl-protein thioesterase 1 (PPT1) was a specific prognostic gene and therapeutic target for mHCC1.

**Conclusion:**

Our study highlighted mechanistic differences among metabolic subtypes and identified potential therapeutic targets for subtype-specific treatment strategies targeting unique metabolic vulnerabilities. The immune heterogeneities across metabolic subtypes may help further clarify the association between metabolism and immune environment and guide the development of novel strategies through targeting both unique metabolic vulnerabilities and immunosuppressive triggers.

## Introduction

Hepatocellular carcinoma (HCC) is the leading cause of cancer-related death in many parts of the world (1). In the past decade, considerable improvements have been made in prevention, surveillance, early detection, diagnosis, and treatment, but HCC remains one of the few cancers with a continued increase in both incidence and mortality (1, 2). The positive results of treatment with sorafenib, a small-molecule multi-kinase inhibitor, in the first-line systemic treatment of advanced HCC, triggered the evaluation of molecular targeted therapy in this disease (3). However, despite several new multi-kinase inhibitors showed survival benefits (4–6), there are still limited therapy options in patients with HCC, with HCC prognosis remaining poor (7, 8). Therefore, an improved understanding of HCC biology that will contribute to expansion of therapeutic arsenal for HCC is clearly and urgently needed.

Cellular metabolism reprogramming is a well-established hallmark of cancer that presents opportunities for cancer diagnosis and therapy (9). Tumors adjust their metabolism to provide sufficient energy and biosynthetic metabolites, which is necessary for malignant cellular proliferation (10, 11). Due to their pivotal cellular functions, recent efforts have sought to devise novel cancer treatments through targeting metabolism vulnerabilities. In addition to metabolism targeted therapy, tumor immunotherapy is raising with both approaches being promising tools to treat tumors (12); in particular, strategies targeting immune checkpoints have shown substantial efficacy in a variety of tumors (13, 14). In HCC, promising responses to immune checkpoint inhibitors PD-1/PD-L1 have been recently reported (15), but similarly to other solid tumors, the response rate was low. Therefore, strategies to improve the efficacy of immune checkpoint inhibitors and to direct individualized medication are urgently required (16, 17).

Since liver is a key organ for whole body energy homeostasis, pathological changes of liver, especially during carcinogenesis, often involve the reprogramming of normal metabolic processes (18). In this scenario, the HCC represents an optimal candidate as a malignancy for developing therapeutics targeting altered metabolism (19). However, HCC is a highly heterogeneous tumor (20). It has not been well established, whether this heterogeneity is caused by distinct metabolic pathways that can be used to stratify HCC into subgroups with clinical significance and biological characteristics (21). Classification of total metabolic characteristics could undoubtedly provide useful information on the metabolic pathways at the systems level of HCC (22). Besides, increasing evidences suggest that alterations in tumor metabolism can also contribute to the inhibition of antitumor response (23, 24). Immunosuppression in the tumor microenvironment is suggested to be based on the mutual metabolic requirements of immune cells and tumor cells (25, 26). Therefore, the investigation of metabolic heterogeneity as well as the association between the tumor metabolism and the tumor immune microenvironment could provide a better understanding of the complex molecular pathogenesis of HCC and lead to discoveries of new possible treatment targets and strategies.

## Methods

### Data resources and reprocessing

The normalized level 3 RNA-sequencing (RNA-seq) data and corresponding clinical information of HCC patients were collected from The Cancer Genome Atlas (TCGA) database portal. RNA-seq data (FPKM values) were transformed into transcripts per kilobase million (TPM) values, which are closer in format to those resulting from microarrays and more comparable between samples. Mutational data for all samples with RNA-seq data available was also downloaded. Furthermore, other HCC datasets were obtained from the GEO public database.

### Consensus clustering for metabolic subtype

Genes belonging to the KEGG subset of canonical pathways were searched in the Molecular Signature Database (MSigDB, https://www.gsea-msigdb.org/gsea/msigdb) (27), and genes involved in the metabolism pathways were collected. Genes displaying a median TPM < 1 across patients were considered extremely low expression and were removed from the consensus clustering analysis. Consensus clustering was performed on metabolic genes using ConsensusClusterPlus R package (parameters: 160 reps=50, pItem=0.8, pFeature=1). The optimum cluster number was determined by testing 2 to 10 clusters, and based on CDF and Δ(K). The collected pathways and their respective genes were subjected clustering analysis are listed in **Supplementary Table S1**.

### Identification of maker genes of HCC subtypes

Hierarchical clustering is used to classify samples into different categories based on the sample’s gene expression matrix, and label different sample categories (28). Taking the expression value of the gene as an input feature and the classification of the sample as an output value, a random forest algorithm was used to construct a prediction model. We used the out-of-bag error rate as an index to evaluate the importance of the features and rank them, and take the first 100 and the first 50 features as the features for subsequent analysis. Scikit-learn was used as a tool for related calculations (29).

### Differentially expressed mRNA analysis

Level-3 RNA-sequencing (RNA-seq) data containing 374 HCC samples and 50 normal controls was downloaded from The Cancer Genome Atlas (TCGA). The differentially expressed genes (DEGs) were identified using the Edge R package for R software. |Fold change| > 2 and adjusted P-values < 0.05 were set as the thresholds.

### Gene set enrichment analysis

The Gene set enrichment analysis (GSEA) was performed to identify enrichment degree of gene set among tumors of a certain metabolic subtype and compared with other samles (27). Significance was considered for values of corrected P as recommended by the software (https://www.gsea-msigdb.org/gsea/downloads.jsp). Gene sets were downloaded from the MSigDB. The GSEA results were merged by using GSVA R package.

### Estimation of metabolic pathways’ activity

Gene Set Variation Analysis (GSVA) is a pathway/gene set-based analysis approach that provides an overall pathway or gene-set activity score for each sample (30). We estimated the activities of metabolic pathways through GSVA. GSVA z-scores for KEGG metabolic gene-sets from the MSigDB and other gene-sets were calculated using the GSVA R package. We considered that the activity a given metabolic pathway was different between two groups if the median of GSVA values across one group differed significantly (Mann-Whitney q-value<0.1) and more than 0.2 from that measured across another group. Besides, we estimated T cell exhaustion scores for each HCC sample by using GSVA and a gene set including signature genes of exhausted CD8+ T cells (31).

### Estimation of the abundance of immune cell populations

GSVA and the single sample Gene Set Enrichment Analysis (ssGSEA) are two most widely used methods that carry out sample level enrichment analysis. Both are unsupervised gene set enrichment methods that compute an enrichment score integrating the collective expression of a given gene set relative to the other genes in the sample. A previous study revealed a significant consistency of the two methods in evaluating immune populations across 28 cancer types (32). Besides, they also compared different signatures identifying immune cell populations reported in previous articles and constructed a new set of gene signatures with better discrimination than that shown in previous sets. According to their experience, we chose GSVA method because its advantage in reducing the noise of the data; the immune signatures were also retrieved from the same publication (32).

### Analysis of PPT1 expression in different cell types in HCC at the single-cell level.

Tumor Immune Single Cell Hub (TISCH, http://tisch.comp-genomics.org), a single cell RNA sequencing (scRNA-seq) application platform that can systematically and comprehensively study the heterogeneity of tumor microenvironment (TME) (33), was used to explore the relationship between PPT1 and the TME on cell level.

### Cell culture

The human HCC cell lines Huh-7 (JCRB0403) and JHH-7 (JCRB1031) were obtained from the Health Science Research Resources Bank (Osaka, Japan). Cells were grown in Dulbecco’s Modified Essential Media (DMEM, Hyclone) supplemented with 10% fetal bovine serum (FBS) and penicillin/streptomycin (Gibco, Grand Island, NY, USA) in a humidified incubator at 37°C in a 5% CO_2_ atmosphere.

### Vector construction and lentivirus transfection

Human pyruvate kinase M2 (PKM2) cDNA was cloned into the pCDH-CMV-MCS-EF1-Neo vector and palmitoyl-protein thioesterase 1 (PPT1) was cloned into the pCDH-CMV-MCS-EF1-Puro vector by IGE (Guangzhou, China). After confirming the PKM2 and PPT1 sequence by sequencing, the plasmid was co-transfected into HEK293T cells (ATCC, RRID: CVCL_0063) with the lentivirus packaging plasmids psPAX2 and pMD2G to produce lentivirus particles. To knock down the PKM2 and PPT1 expression, the shRNA sequence of PKM2 was cloned into the pLKO.1-Neo lentiviral vector and the shRNA sequence of PPT1 was cloned into the pLKO.1-Puro lentiviral vector by IGE (Guangzhou, China). The oligonucleotide sequences of shRNAs are listed in **Supplementary Table S5**. Similarly, the lentiviral vector was transfected into HEK293T cells together with packaging vectors pMD2.G and psPAX2 to produce lentivirus particles. The media containing retroviruses were collected 72h after transfection, centrifuged to remove cell debris, and then filtered. The lentivirus particles were then used to transduce Huh-7 and JHH-7 cells. Control cells were transfected with a control empty vector.

### Western blotting

Total protein was extracted by using RIPA lysis buffer containing a cocktail of protease and phosphatase inhibitors. Using the BCA Protein Assay Kit (CWBio, China, Cat#CW0014S), the concentration of the protein was determined. Following the previous process, 20 μg total proteins extracted from Huh-7 cells were separated electrophoretically in 10% SDS polyacrylamide gels. Separated proteins are transferred to polyvinylidene fluoride (PVDF) membranes. The membrane was then blocked with 5% BSA at room temperature for 1h before being incubated with the primary antibodies overnight at 4°C. Next day, the membranes were incubated for 1h at room temperature with secondary antibodies. Eventually, the immunoblotting was tested using the ECL Chemiluminescence Kit (Thermo Fisher Scientific, USA, Cat#32109). The antibodies are shown in **Supplementary Table S4**.

### Cell viability assay

MTT assay was used to evaluate cell viability. Cells were seeded in 96-well plates (2 × 10^3^/well) (in triplicate for each condition) and were cultured for different hours (34). The 20 µl MTT (Sigma, Saint Louis, USA) stock solution (5 mg/ml) was added to each well and incubated for 2h, the absorbance was measured at a wavelength of 570 nm.

### Glycolysis stress tests (ECAR)

ECAR was estimated by using Seahorse assays with a Seahorse XFp Analyzer, 2.0 × 10^4^ Huh-7 cells per well were plated onto 8-well plates and glycolysis stress test was performed following the manufacturer’s specifications (35, 36). The assay DMEM media was free of glucose and pyruvate. The concentration of drugs used for test were as following: glucose (10.0 µM), oligomycin A (1.0 µM), and 2-deoxy-D-glucose (2-DG, 100 µM).

### Lactate production assay

5 x 10^5^ HCC cells were incubated in phenol red-free medium at 37°C, 5% CO_2_ for 1h. The supernatant medium was collected. The lactate secreted by the cells was measured using the Lactate Colorimetric/Fluorometric Assay Kit according to the manufacturer’s protocol (BioVision, Inc.) (37).

### Statistical tests

All quantitative data were conducted at least three independent experiments and expressed as the mean ± SD. For comparisons between two groups, statistical significance for normal distribution data was estimated by unpaired Student t-test, otherwise Mann-Whitney U test was applied. For comparisons of more than two groups, One-way ANOVA multiple comparison was used. Correlation coefficients were computed by Spearman and distance correlation analyses. The influence of a single factor on the survival was evaluated through the Cox proportional hazard model, Kaplan-Meier survival curves, or Log-rank test. All statistical analyses were conducted using R (https://www.r-project.org/) or SPSS software (version 17.0). Two-sided P values of less than 0.05 were considered statistically significant.

## Results

### Classification of metabolic subtypes in HCC

**Figure 1:**
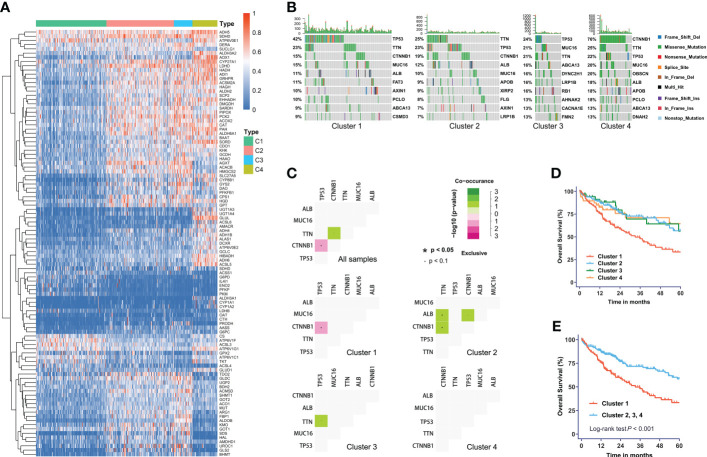
The metabolic subtypes in HCC. **(A)** Unsupervised clustering of HCC samples for 371 HCC patients in the TCGA cohort. **(B)** Correlation between the distribution of highly variant mutated genes and metabolic subtypes. **(C)** Co-occurrence analysis of mutations in whole cohort and each subtype. **(D)** Kaplan-Meier curves with univariate analyses of overall survival in patients stratified by metabolic subgroup. **(E)** Kaplan-Meier curves with univariate analyses of overall survival in patients of cluster 1 versus those of other three clusters. Log-rank test P values are shown.

We obtained four robust subtypes of HCC in TCGA dataset (**Supplementary Table S2**). A heatmap based on one hundred genes obtained with machine learning was shown in **Figure 1A**. The robust classification revealed that HCC tumors displayed highly heterogeneous expression of genes that were directly involved in metabolism. The mHCC1 was defined as the largest group (144/371; 38.8%), followed by mHCC2 (138/371; 37.2%), mHCC4 (51/371; 13.7%), and mHCC3 (38/371; 10.2%). These subtypes are henceforth termed mHCC1 (cluster 1), mHCC2 (cluster 2), mHCC3 (cluster 3), and mHCC4 (cluster 4). We noted distinct patterns of gene mutations across the four different metabolic subtypes (**Figures 1B, C**). For example, mutations in TP53 were enriched in mHCC1, and 76% of MHCC4 tumors showed mutations in CTNNB1. In addition, co-occurrence analysis using the five most frequent mutations in HCC indicated different relationships among the five common mutations across four subtypes. A mutual exclusion between TP53 mutations and CTNNB1 mutations in mHCC1 tumors was observed, while co-occurrence between TTN mutations and mutations in CTNNB1 or ALB were found in mHCC2. These results indicated that there are multiple metabolic phenotypes in HCC driven by different oncogenic mutations. The mHCC1 was associated with the shortest survival while the prognosis of other three subtypes were similar (**Figures 1D, E**).

### Landscape of functional annotations across four subtypes

**Figure 2:**
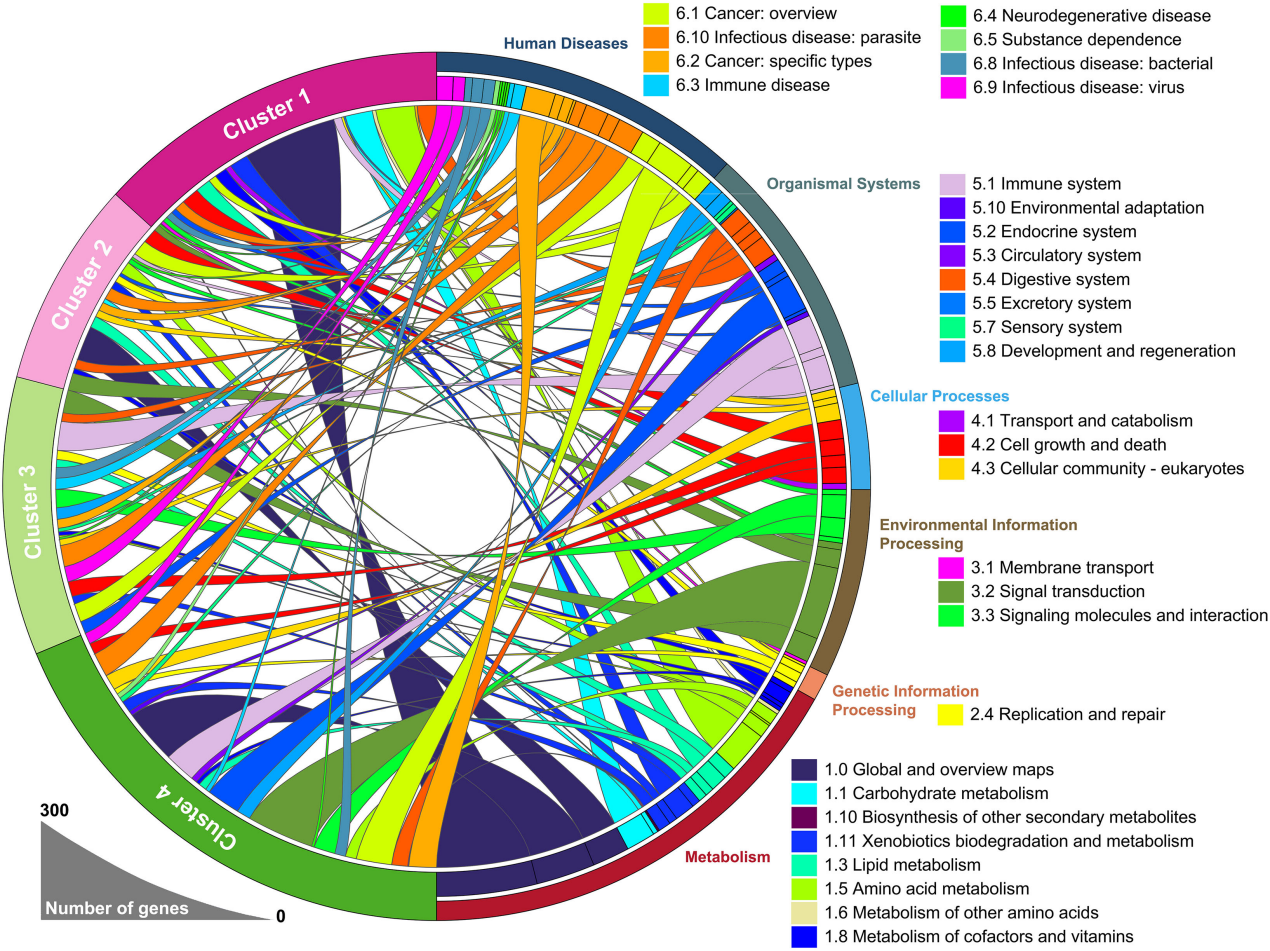
A circular plot showing the differentially expressed genes and their associated biological processes. The differentially expressed genes in each group were calculated separately, using 50 normal liver samples as control group.

To identify the underlying biological functional characteristics of each subtype, signature genes in each group were collected. KEGG analysis of the signature genes was conducted and the results were visualized in **Figure 2**. Among those signature genes dysregulated in each subtype/cluster, we identified that the mHCC1 had much larger proportion of genes enriched in metabolism-related pathways than that in other clusters; especially including “carbohydrate metabolism”, “lipid metabolism”, and “amino acid metabolism”, which were major reprogrammed metabolic pathways in malignant cells. In addition, we noted that most genes that were enriched in “signal transduction”, “signaling molecules and interaction”, and “cellular community” came from mHCC4, followed by mHCC2 and mHCC3, but barely from mHCC1. Besides, immune-related pathways were more likely to be enriched by genes from mHCC3 and mHCC4, such as “Immune system”, “Infectious disease: bacterial”, “Infectious disease: viral”, “Infectious disease: parasite”, and “Immune disease”.

### Association of different subtypes with metabolic pathway profiles

**Figure 3:**
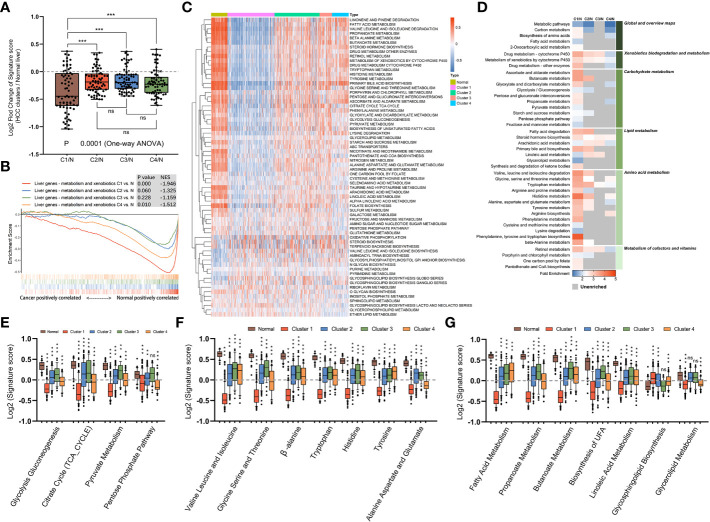
Metabolic landscape across metabolic subgroups. **(A)** In samples of normal livers and each subtype, the GSVA values of 69 KEGG metabolic pathways were measured respectively. With the normal liver as the control group, fold changes of GSVA values in each subtype were obtained and shown in a box plot. Each dot represents the fold change in GSVA value of one metabolic pathway between a subtype and the control group. **(B)** Gene set enrichment analysis of a gene sets including metabolic genes in healthy liver, with all transcripts ranked by the log2 (Fold Change) between clusters and normal liver. **(C)** Heatmap depicting GSVA values of metabolic pathways across each subgroup. **(D)** Heatmap showing the results of KEGG clustering results based on differentially expressed genes in each subgroup. **(E–G)** The GSVA values of pathways involved in glucose metabolism **(E)**, amino acid metabolism **(F)**, and fatty acid metabolism **(G)** for samples in each subgroup were shown in box plots. (ns: not significant, **: P < 0.01, ***: P < 0.001).

We next supposed that subtypes produced through gene clustering had specific features in different metabolic pathways and employed a sample-level gene set enrichment method (GSVA) to compute the GSVA enrichment scores of the selected metabolic pathways. The differences in GSVA scores for each pathway between normal liver and four subtypes were calculated. As shown in **Figure 3A**, compared with other subtypes, mHCC1 displayed significantly dysregulated fold change in GSVA scores with reference to those in normal livers. It was well known that HCC cells are metabolically distinct from normal hepatocytes and express different metabolic enzymes (38, 39). Our next gene set enrichment analysis (GSEA) using a gene set involving metabolic genes of normal liver revealed a significant absence of normal metabolic genes in samples of mHCC1 and mHCC4, but no significant differences in mHCC2 and mHCC3 (**Figure 3B**). These results were consistent with the KEGG analysis visualized in **Figure 2** and suggested different types of metabolism impairments among subtypes. Similarly, as shown in **Figures 3C, D**, there was a remarkable difference between GSVA scores for most metabolic pathways of the mHCC1 and other three clusters as well as normal liver samples; in contrast, GSVA scores of the most pathways in both mHCC2 and mHCC3 showed limited differences compared with normal liver samples, which is consistent with the GSEA results.

We next compared GSVA scores of major metabolism reactions that are critical to carcinogenesis, including carbohydrate metabolism (**Figure 3E**), amino acid metabolism (**Figure 3F**), and lipid metabolism (**Figure 3G**). Overall, compared with normal liver samples, tumors in mHCC1 were most significantly differentially enriched for almost all the selected pathways. The GSVA enrichment pattern in different pathways of carbohydrate metabolism was identical, mHCC1 had most significant differences in GSVA scores than in normal liver, followed by mHCC4, while mHCC2 and mHCC3 had similar scores compared with each other. In the respect of amino acid metabolism and lipid metabolism, mHCC2, mHCC3, and mHCC4 had similar enrichment in many pathways but mHCC1 still showed a significantly differential enrichment for all pathways. Specifically, mHCC4 displayed much more significant enrichment in “Pentose phosphate pathway” than other clusters and showed a similar enrichment in both “Alanine aspartate and glutamate pathway” and “Glycolipid metabolism pathway” compared with mHCC1. In addition to the characteristic metabolic pathways specifically present above, there are some amino acid metabolic pathways that are significantly reduced in mHCC1.

### Analysis of differences in specific metabolic pathways across four subtypes

**Figure 4:**
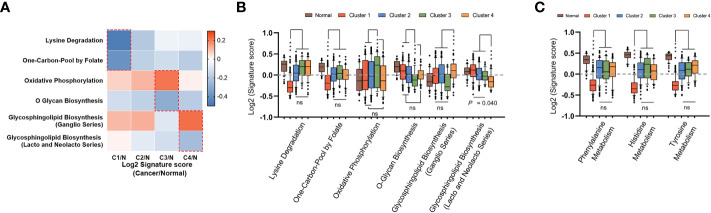
Identification of unique metabolic characteristics in each subtype. **(A)** Heatmap displaying the GSVA score of which pathway has exclusivity significant difference between a subtype and normal control group. Pathways that showed significant difference in only one group were marked with dotted box, and were shown in box plots of **(B, C)**, respectively. (ns: not significant, ***: P < 0.001).

To complete the landscape of the metabolic pathways across the four subtypes, we further evaluated whether each subtype had unique metabolic pathways enriched. If a pathway was especially different (Mann-Whitney q-value<0.05 and difference between medians of GSVA was more than 0.2) in only one cluster, it was regarded as a characteristic metabolic pathway in corresponding subtype. As shown in **Figures 4A–C**, there were six characteristic pathways in total identified in mHCC1, mHCC3, and mHCC4. “Lysine Degradation” and “On-Carbon-Pool by Folate” were identified as characteristic metabolic pathways of mHCC1, “Oxidative Phosphorylation” and “O-Glycan Biosynthesis” in mHCC3, and two sub-pathways of “Glycosphingolipid Biosynthesis” in mHCC4, while none in mHCC2. In addition to the characteristic metabolic pathways specifically present above, there are some amino acid metabolic pathways that are significantly reduced in mHCC1.

### Identification of survival signatures and its heterogeneity across metabolic subtypes

**Figure 5:**
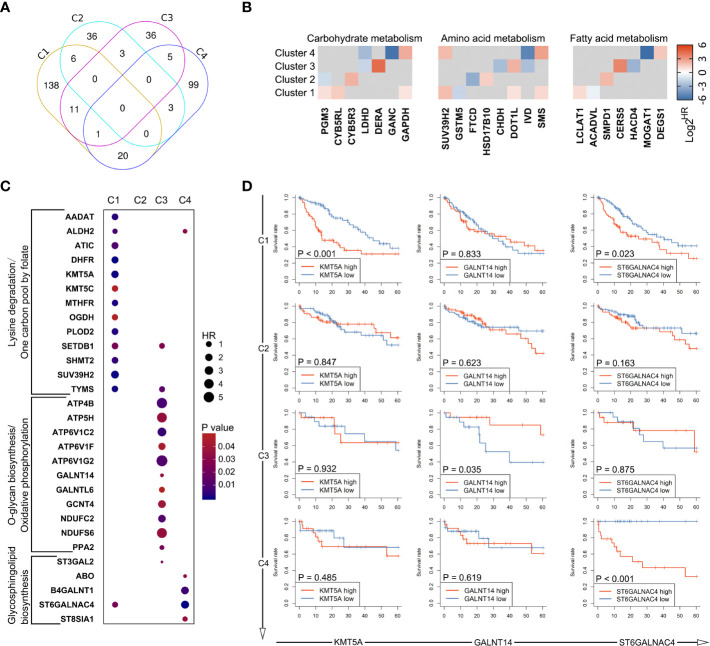
Heterogeneity in prognostic significance of metabolic genes stratified by the metabolic subtypes. **(A)** A venn diagram showing common and specific prognostic genes across four subtypes. **(B)** The hazard ratio values (HRs) of prognostic genes involved in glucose metabolism, amino acid metabolism, and fatty acid metabolism are depicted in heatmap. Only HRs of genes with significant prognostic significance are shown. **(C)** Dot plot depicting the prognostic genes involved in unique metabolic pathways described in **Figure 4B**. **(D)** Several genes were selected from **(C)**, including KMT5A, GALNT14, and ST6GALNAC4. Survival curves showing the predictive role of these genes for over survival in each subtype.

The above observations led us to question how these differences among metabolic subtypes relate to clinical outcome of the patients. Therefore, the prognostic value of metabolic genes in each subtype was investigated using log-rank test and univariate Cox regression model. The results of survival analysis are summarized in **Supplementary Table S3**. Remarkably, only a small portion of metabolic genes with prognostic significance were shared by multi-subtypes, and none was shared by all four subtypes (**Figures 5A, B**), suggesting distinct role of genes across subtypes even for the same metabolic pathway. Furthermore, survival analysis based on genes involved in the six characteristic metabolic pathways also revealed specific prognostic value of metabolic genes in their corresponding clusters (**Figures 5C, D**). These data suggested subtype-specific roles for these unique tumor metabolic pathways as mechanisms contributing to tumor progression and identified some genes of unique metabolic pathways as potential targetable metabolic vulnerabilities in subtype-specific manner.

### Relevance of metabolic clusters in immune features

**Figure 6:**
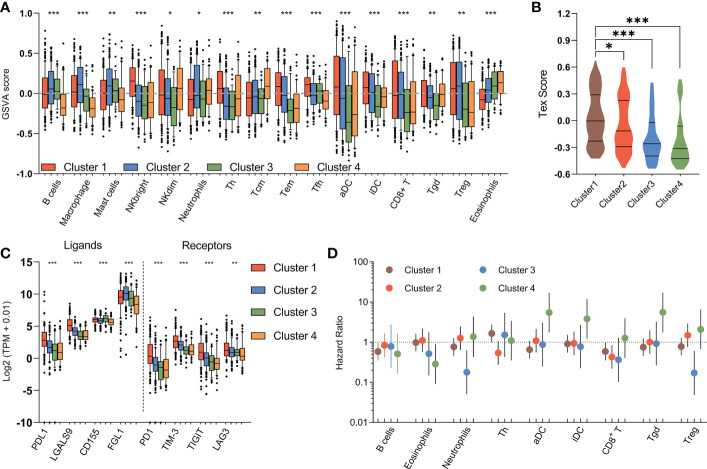
Immune characteristics of metabolic subtypes. **(A)** Box plots depicting the abundance of immune cell populations in each subgroup. **(B)** Violin plots showing the distribution of T cell exhaustion scores (Tex Score) in samples of each group. **(C)** Box plots depicting the expression of immune checkpoints across metabolic subtypes. **(D)** The prognostic significance of the abundance of each immune cell population in each subtype are summarized in a forest plot. (*P < 0.05, **P < 0.01, ***P < 0.001).

The immune response is associated with dramatic modifications in tissue metabolism, and emerging evidences suggested the tremendous impact of tumor metabolic reprogramming on the immune response (40). Therefore, we assessed the relationship between the metabolic subtypes and previously defined scores which indicating the abundance of various immune cell populations. The immune landscape revealed significant intra-cluster heterogeneity (**Figure 6A**). Consistent with an immune-cold phenotype, tumors in mHCC3 had the lowest rate of leukocytes infiltration. Contrary to the immune-cold mHCC3, tumors in mHCC1 had the highest cell infiltration fraction and it was mainly characterized by higher infiltration of lymphocytes. Interestingly, although tumors in mHCC1 displayed the highest infiltration of cytotoxic cells including CD8+ T cells, Tgd, and NK cells, mHCC1 was the subtype with both poorest prognosis and highest T cell exhaustion score (**Figure 6B**), suggesting that the tumor microenvironment of mHCC1 was immune-hot but highly immune-exhausted type. Most suppressive ligands and receptors for immune checkpoint showed the highest expression levels in mHCC1 (**Figure 6C**), which is consistent with an immune-suppressive phenotype. Emerging experimental data indicated that the presence of a pre-existing intra-tumoral T cell infiltration, checkpoint molecules (PD-1, PD-L1 expression) could favor a clinical response (41), therefore, we hypothesized that this subtype could benefit much from immune therapy only if the immunosuppressive situation was relieved.

Different from the extreme low/high immune infiltration shown above for mHCC3 and mHCC1, mHCC2 demonstrated a more balanced and favorable immune profile. One major difference from mHCC1 is that the immune composition in mHCC2 was enriched with B cells, macrophages, mast cells, and neutrophils, suggesting an immune microenvironment tended to towards innate immunity and inflammation. The remaining mHCC4 were more diverse with intermediate levels of immune features. The mHCC4 had high infiltration levels of NKdim, Th, Tcm, and Tgd, but showed a low level of other lymphocytic cells, and extremely low levels of B cells, macrophages, mast cells, and neutrophils, which was opposite to mHCC2. Additionally, mHCC4 had the highest level of eosinophils. Together with the specificity of eosinophils infiltration as an index for good prognosis in mHCC4, the above findings supported the major anti-tumor role of eosinophils in the metabolic subtype mHCC4.

The prognostic role of immune cells shown by survival analysis between inter-and intra-cluster was in a high degree of heterogeneity (**Figure 6D**). For example, among the important cytotoxic T cells, such as CD8+ T cells, and Tgd cells, we found that increased rate of CD8+ T cell infiltration was associated with good prognosis in mHCC1, mHCC2, and mHCC3, but it was not linked with prognosis in mHCC4; in contrast, Tgd infiltration was associated with significantly poor prognosis in mHCC4, but showed no correlation with prognosis in other three clusters. Meanwhile, some immune cells had the opposite prognostic role in different clusters, such as that infiltration of Treg cells was a poor factor in the prognosis of mHCC4 but a good factor of prognosis in mHCC3. The prognostic heterogeneity of immune infiltration revealed different roles of immune cells in different HCC subtypes. Since the metabolic subtypes were produced based on metabolism genes that did not involve signature genes of immune cells, these results suggest a high heterogeneity in component and function of immune microenvironment across these four metabolic subtypes, and support a combination treatment strategy targeting metabolism and immune microenvironment, or the attempt to dig out new target of dual function that combined metabolism-regulation as well as immune-modulation.

### Identification of palmitoyl-protein thioesterase 1, a metabolic gene, as a therapy target for T cell exhaustion with metabolic subgroup specific

**Figure 7:**
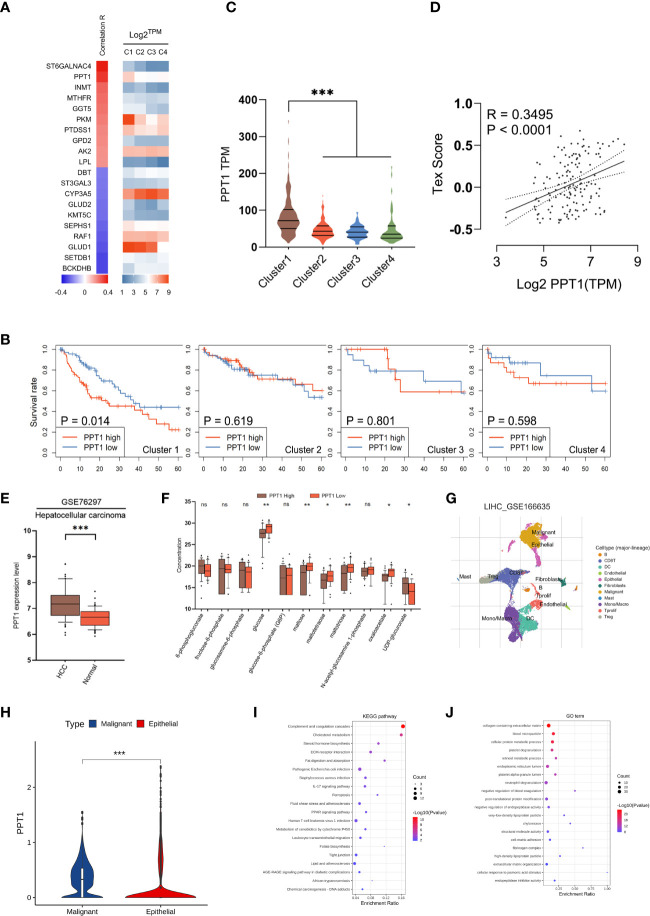
Identification of PPT1 as a therapy target. **(A)** Heatmap showing the correlation between prognostic metabolic genes and Tex score. The top 10 most positively/negatively correlated genes are shown. **(B)** Survival curves showing the distinctive prognostic value of PPT1 in different metabolic subtypes. **(C)** Violin plots showing the expression of PPT1 in samples of different subtypes. **(D)** Scatter plot depicting the correlation between PPT1 gene expression and Tex score. **(E)** PPT1 expression in HCC and non-tumorous tissues was analyzed using data in the LIHC_GSE76297. **(F)** Box plots depicting the glycolytic related metabolites between low and high PPT1 expression subgroup. **(G)** Single-cell RNA sequencing revealed the cell types and distribution of HCC. **(H)** Violin plots showing the PPT1 expression between malignant and epithelial cells. **(I, J)** The KEGG pathways **(I)** and GO biological processes **(J)** enrichment analysis of PPT1 in malignant cells. (ns: not significant, *: P < 0.05, **: P < 0.01, ***: P < 0.001).

Considering that mHCC1 had the poorest prognosis, we tried to identify new therapeutic targets for this subtype. The prognostic metabolic genes were identified, and their correlation with T cell exhaustion, the important feature of mHCC1, was investigated (**Figure 7A**). Of genes most closely related with T cell exhaustion, we identified palmitoyl-protein thioesterase 1 (PPT1), which was specifically upregulated in mHCC1 and was a specific prognosis factor for mHCC1 (**Figures 7B, C**). The positive correlation between PPT1 and Tex score (**Figure 7D**, R = 0.3495, P<0.0001) suggested that PPT1 might contribute to T cell exhaustion. To determine the relationship between PPT1 and glucose metabolism, we analyzed the data of Transcriptomics and metabolomics in the study of Chaisaingmongkol et al. (42). Data LIHC_GSE76297 was used to confirm the high expression of PPT1 in HCC tumor tissues. Through the analysis of glucose metabolism-related metabolites, the content of glucose, maltose and maltotriose decreased in the high expression group of PPT1, suggested that the level of glycolysis was exuberant in tissues with high expression of PPT1 (**Figures 7E, F**). Then, we analyzed the data LIHC_GSE166635 in GEO database using TISCH platform, and the results showed that the expression of PPT1 in malignant cell was strikingly higher than that in epithelial cells (**Figures 7G, H**). To verify the biological function and mechanism of PPT1, functional annotation analysis was performed on PPT1 in malignant cell. The KEGG pathways and GO biological processes enrichment suggested that PPT1 was closely related to tumor metabolism, immunity, tumor microenvironment and extracellular matrix (**Figures 7I, J**), which was consistent with the above results that PPT1 is related to tumor metabolism and immunosuppression (43, 44).

**Figure 8:**
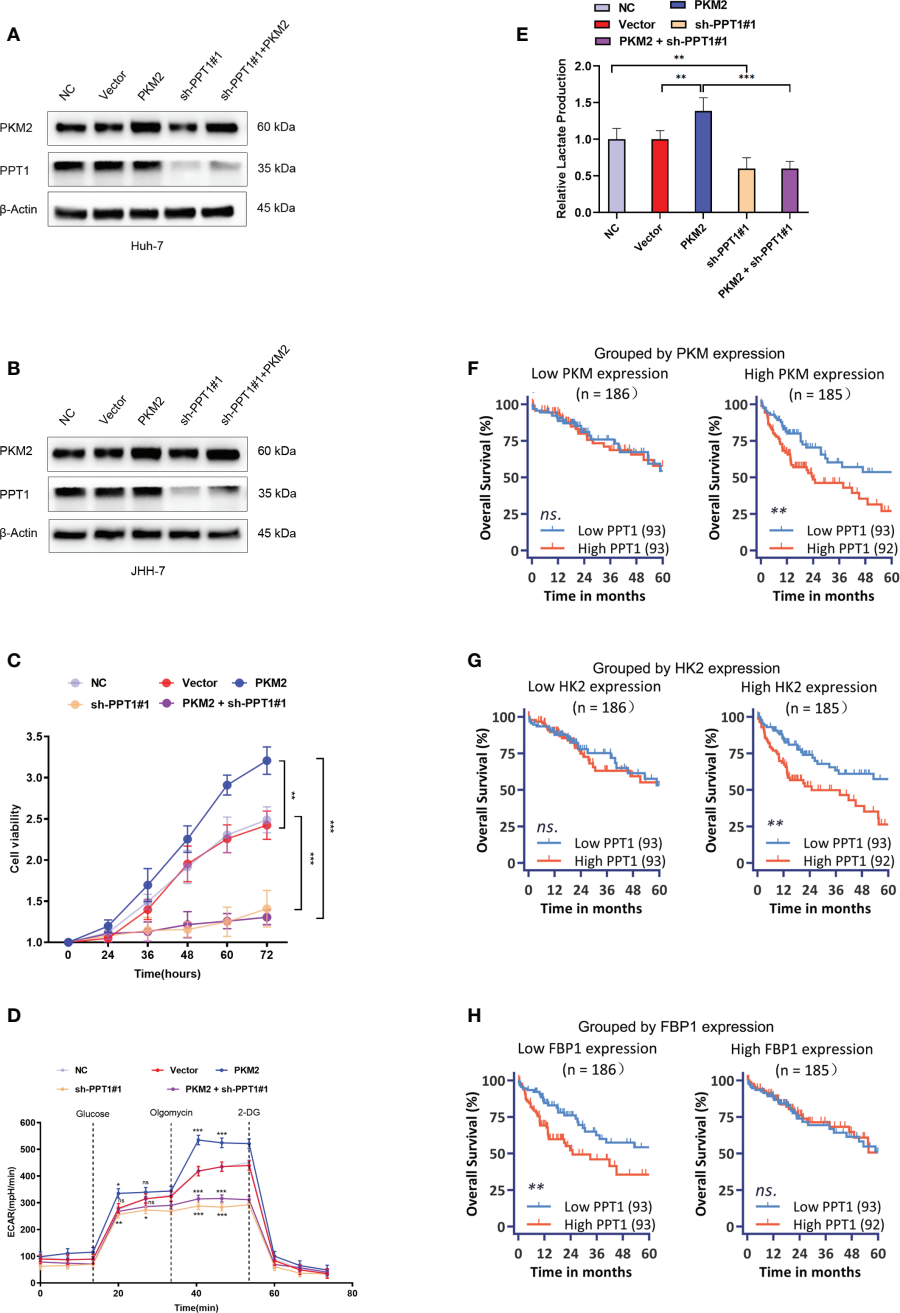
Effect of PPT1 knockdown on human HCC cells. **(A, B)** Stable knockdown of PPT1, and overexpression of PKM2 in Huh-7 **(A)** and JHH-7 **(B)** cells by lentiviral vector transfection. The knockdown effect on protein levels were shown for normal control cells (NC), cells transfected with empty vector (Vector), PPT1 knockdown cells (sh-PPT1#1), PKM2 overexpression cells (PKM2), and combination of PKM2 overexpression and PPT1 knockdown (PKM2+ sh-PPT1#1). β-Actin was used as a loading control. **(C)** *In vitro* cell proliferation curves showing the effect of PKM2 overexpression, PPT1 knockdown, or in combination of PKM2 overexpression and PPT1 knockdown on huh-7 cells. **(D)** Seahorse metabolic analysis (ECAR) of normal control cells (NC), cells transfected with empty vector (Vector), PPT1 knockdown cells (sh-PPT1#1), PKM2 overexpression cells (PKM2), and combination of PKM2 overexpression and PPT1 knockdown (PKM2+ sh-PPT1#1). **(E)** Relative lactate production from hun-7 cells upon treatment as annotated. **(F–H)** Survival curves showing the predictive role of PPT1 for prognosis stratified by PKM2 expression **(F)**, HK2 expression **(G)**, or FBP1 expression **(H)**. The error bars are expressed as the mean ± SD of three independent experiments. (ns: not significant, *: P < 0.05, **: P < 0.01, ***: P < 0.001).

In a very recent study, Rebecca et al. identified PPT1 is the molecular target of a novel chloroquine derivative, the DC661; they found that knockout of PPT1 in several cancer cell lines using CRISPR-Cas9 editing resulted in significant impairment of tumor growth similar to that observed with DC661 treatment, which supported the tumor driver role of PPT1 in cancer (45, 46). Therefore, a further question was raised whether PPT1 as a therapeutic target had subtype-specific property and immunomodulating function. *In vitro* study was carried out to verify the above questions. We enhanced the glycolysis activity through overexpression of PKM2 in Huh-7 and JHH-7 cells, cell lines with the intermediate glycolysis level. Downregulation of PPT1 significantly neutralized the proliferative advantage of PKM2 overexpression cells (**Figures 8A–C**). In addition, PKM2 overexpression markedly increased ECAR (**Figure 8D**), and we found downregulation of PPT1 in PKM2-overexpressed cells induced a larger drop in ECAR than it did in normal cells, and the same was observed for the lactate production ability (**Figure 8E**), which partly explained the positive correlation between PPT1 expression and Tex score. We next divided TCGA samples into two groups based on the expression of PKM2, HK2, or FBP1, respectively. Interestingly, the prognostic value of PPT1 expression was only significant in TCGA samples with higher expression of PKM2 (**Figure 8F**) or HK2 (**Figure 8G**), two enzymes promote glycolysis, or in TCGA samples with lower expression of FBP1 (**Figure 8H**), an enzyme inhibits glycolysis.

### PPT1 promotes the proliferation, migration, and invasion of HCC *in vitro* and vivo.

**Figure 9:**
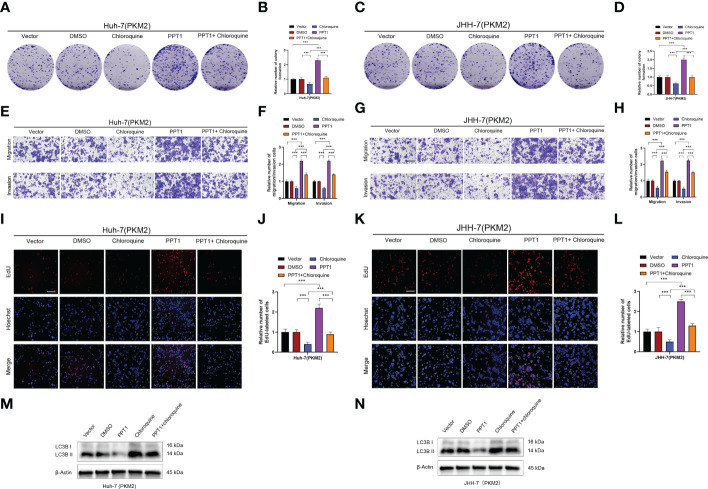
PPT1 promotes the proliferation, migration and invasion of HCC *via* lysosomes *in vitro*. **(A–D)** The colony formation was counted in stable overexpression of PKM2 between Huh-7 **(A, B)** and JHH-7 **(C, D)** cells. **(E–H)** Representative images and histogram analysis of Transwell migration and Matrigel invasion assays in stable overexpression of PKM2 between Huh-7 **(E, F)** and JHH-7 **(G, H)** cells. Scale bars: 100 μm. **(I-L)** Representative images and histogram analysis of EdU assays in stable overexpression of PKM2 between Huh-7 **(I, J)** and JHH-7 **(K, L)** cells. Scale bars: 100 μm. **(M, N)** Western blotting analysis of PPT1 and LC3B in stable overexpression of PKM2 between Huh-7 **(M)** and JHH-7 **(N)** cells. β-Actin was used as a loading control. The error bars are expressed as the mean ± SD of three independent experiments. (***: P < 0.001).

Recently, it has been reported that PPT1 is essential for the function of lysosomes, which, as the center of cellular energy sensing and metabolic regulation, play a driving role in the malignant progression of tumor cells (45). In HCC, the increase of glycolysis level and the further enhancement of lysosome and autophagy activity lead to poor prognosis of patients (47). PKM2 is a key rate-limiting enzyme in glycolysis and an important factor in HCC metabolism (48). Therefore, we decided to find out whether PPT1 depends on PKM2 to promote tumor progression through autophagy. Chloroquine, a lysosomal autophagy inhibitor, was used to inhibit autophagy. In Colony formation assay (**Figures 9A–D** and **Supplementary Figures S1A–D**), Transwell assay (**Figures 9E–H** and **Supplementary Figures S1E–H**) and EdU assay (**Figures 9I–L** and **Supplementary Figures S1I–L**), compared with the stably knockdown PKM2 cells, PPT1 could significantly promote the proliferation, migration and invasion of HCC tumor cells in stable overexpression of PKM2 between Huh-7 and JHH-7 cells, interestingly, the proliferation, migration and invasion of tumor cells decreased strikingly after the treatment of chloroquine. Furthermore, Western blotting analysis showed that under the condition of high expression of PKM2, PPT1 could significantly affect the expression of autophagosome protein marker LC3B, while the accumulation of LC3B -II expression increased after chloroquine was added, suggesting that the lysosomal function might be impaired (**Figures 9M, N**). Collectively, PPT1 may be dependent on PKM2 to promote the progress of HCC through the autophagy.

**Figure 10:**
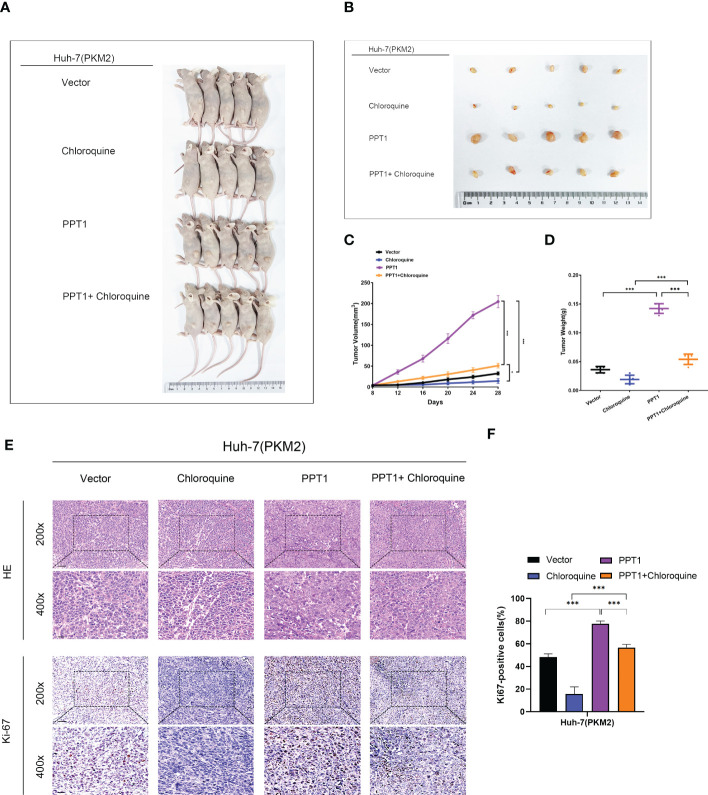
PPT1 promotes HCC tumorigenicity and progression *in vivo*. **(A, B)** Representative images of subcutaneous xenograft tumors in stable overexpression of PKM2 Huh-7 cells treated with Vector, Chloroquine, PPT1 lentivirus and PPT1 lentivirus + Chloroquine (n=5). **(C, D)** The tumor volume **(C)** and weight **(D)** were evaluated in each group. **(E, F)** Representative images **(E)** and histogram analysis **(F)** of IHC for Ki-67. Scale bars: 200× =50 μm; 400× =20 μm. The error bars are expressed as the mean ± SD of three independent experiments. (*: P < 0.05, ***: P < 0.001).

To further verify the role of PPT1, we constructed Mouse subcutaneous xenograft models. Compared with the control group, the injection of Huh-7 cells stably overexpressing PPT1 and PKM2 significantly promoted tumor growth, while after treatment with chloroquine, tumor growth was inhibited to some extent (**Figures 10A–D**). In addition, IHC staining showed that Ki-67 levels were notably increased in the tissues of mice treated with stable overexpression of PPT1 and PKM2, suggesting that tumor proliferation was obvious (**Figures 10E, F**). Taken together, PPT1 relies on PKM2 to promote the malignant progression of HCC by mediating autophagy.

## Discussion

HCC is highly heterogeneous and thus difficult for treatment cancer (49). In this regard, continuous progress in the understanding of molecular tumor subtypes is needed to accelerate the development of personalized treatment for HCC patients. Our study established four HCC subtypes with distinct profiles based on the expression of metabolic genes. We found distinct differences in metabolic pathways, immune profiles, and in clinical survival between four major HCC subtypes. Moreover, the markedly immune heterogeneity among the metabolic subtypes provides more biological and clinical significance to our classifier and suggests subtype-specific therapeutic strategies targeting metabolic dependencies and immune regulators alone each or in combination.

Clear evidences supported that metabolic alterations are common for all tumor types (50). As a key organ for whole body energy homeostasis, the liver carries out many metabolic functions (51). Consequently, it is not surprising that metabolic reprogramming is critical during HCC carcinogenesis. Our study demonstrated that HCC was characterized by the absence of normal metabolic genes in noncancerous liver, which was more evident in individual subtypes. This result suggested that alterations in the expression of genes involved in metabolism of normal liver contributed to HCC metabolic heterogeneity. An enzyme or a metabolic pathway enriched in HCC and not in the corresponding normal liver tissue could be used to selectively target tumor cells. Therefore, the degree of normal metabolic genes loss seems an indicator for the success of therapies targeting metabolism.

Among all four subtypes, the first subtype (mHCC1) demonstrated the worst prognosis, significant downregulation of metabolic genes enriched in normal liver, and broad alternations in most metabolic pathways. HCC involves multiple metabolic abnormalities. In addition to unique metabolic features, alternations in glucose metabolism, lipid metabolism, and amino acid metabolism attracted much wider attention over the past few years (52–54). Most cancer cells reprogram cellular glucose metabolism to constitute a selective advantage for proliferation (55). Among them, HCC probably displays the most comprehensive reprogramming of glucose metabolism, which was essential for the maintaining of tumor growth and progression (56). Altered amino acid metabolism and lipid metabolism also characterize HCC compared with other liver diseases. GSVA analysis indicated that tumors of the first subtype had significant alternations in all pathways associated with glucose metabolism, and most pathways involved in lipid and amino metabolism in comparison with other tumors and normal liver tissues. This finding suggests that tumors in this subtype rely heavily on reprogramed metabolism, which is therefore an appropriate candidate for metabolism-target therapy.

One of the clinical-translational relevance of this subtype classification is that it suggested that different metabolic subtypes have different therapeutic and prognostic targets, since the prognostic significance of a particular metabolic gene could be different between each subtype. For example, PPT1, a gene that was detected as a special prognostic factor only for mHCC1, regulates the lysosomal acidity necessary for cellular catabolism. The catabolic function and nutrient sensing activity of the lysosome make it as a metabolic signaling center (57). Lysosome is a key central delivery port for substrates destined for breakdown and serve to recycle the constituent building blocks (58). The central position of the lysosome system in cancer have made it a promising target in anti-tumor therapy, especially for those alterations that are highly dependent on metabolism to fulfill their anabolic demands (46). A previous study has identified that KO PPT1 cells showed impaired lysosomal deacidification, decreased proliferation, and increased apoptosis both *in vitro* and *in vivo* (45). Consistent with the above assumption, we found PPT1 have special prognostic value in the first subtype, and knockdown of PPT1 significantly neutralized the PKM2 overexpression-induced growth advantage in HCC cells. Despite PPT1 being a promising target for HCC tumors with relatively abundant metabolism alternations, these findings supported that the therapy strategy in a metabolic subtype-specific manner might be more efficient than those trying to cover all patients. The subtype classification of this study provided clues for future research.

In healthy tissues, neighboring cells cooperate to build a harmonious metabolic environment, which is usually disrupted in cancer tissues (59). As a result of altered tumor metabolism, cells in tumor microenvironment suffer from lacking essential nutrients while atypical metabolites accumulate, along with impaired antitumor immunity (40, 60, 61). One example is the competition for glucose between T lymphocytes and tumor cells; T cells consume extracellular glucose to fulfill their activation, and suppression of glucose take up, such as knockout glucose transporters, inhibited proliferation of activated CD4+ T cells and generation of effector T cells (62). In turn, elevated lactate production in glycolytic cancer cells suppressed survival of effector T and NK cells and promoted immune escape (63). In this study, perhaps surprisingly, we found distinct immune infiltration profiles, as well as expression of immune checkpoints and T cell exhaustion levels in different metabolic subtypes. Additionally, the prognostic significance of infiltration level for the same immune cell was distinct in different subgroups. These findings revealed an intimate link between metabolic subtypes and immune heterogeneity; suggested personalized immunotherapy strategy according to the metabolic subtypes. For example, the mHCC1, despite its significantly altered metabolic pathways, was characterized by high immune infiltration, high T cell-exhausted signature, and high expression of suppressive immune checkpoints; all indicating the applicability of treatment strategy through combination of metabolism-targeted and immune-targeted therapies in this subtype. In contrast, mHCC2 was the only subtype which did not show significantly altered expression of normal metabolic genes in the GSEA analysis, suggesting that metabolism-targeted therapy is not a priority for patients with this subtype.

Tumors in mHCC4 had distinct mutations compared with other three groups. More than 76% of patients with this subtype harbored mutations in CTNNB1. A very recent report discovered that Wnt/CTNNB1 mutations could characterize the immune excluded class (cold tumors) and might represent the biomarkers predicting resistance to immune checkpoint inhibitors (64, 65). Consistent with this report, subtype 4 showed a low enrichment level for signatures of most immune cells. Only in subtype 4, CD8+ T cell infiltration had almost no correlation with the prognosis. Besides, many immunosuppressive checkpoints, including PD1, PD-L1, TIM-3, LAG3, and others, showed the lowest expression levels in this subtype. All these suggested innate resistance to checkpoint inhibitors. But fortunately, tumors of this subtype demonstrate highly altered metabolism which is second only to subtype 1. Therefore, effort to uncover new metabolism-related targets in this subtype might be helpful. Additionally, unlike the other three subtypes, increased infiltration of aDC, iDC, or Tgd were all associated with poor prognosis. The unique roles of these immune cells and their correlation with metabolism merits further study.

Another interesting finding in the present study was that some molecules might be good target to “kill two birds with one stone”. For example, PPT1 showed much more correlation with cell viability, glucose metabolism activity, lactate production, and T cell exhaustion in HCCs with enhanced metabolic alterations, which should be especially applicable for the mHCC1 tumors with obvious alterations in metabolism and enhanced proliferation. This also provided clues for the treatment strategy for other subtypes. The tumors in subtype 3 also seemed like “cold’ tumors”; they had aberrant decreased enrichment of immune signatures and showed a low level of metabolism alteration. However, the mHCC3 had its own unique characteristic: CTNNB1 mutations was rare in this subtype; it was the only subtype that benefited from enrichment in aDC; moreover, CD8+ T cell enrichment showed the lowest HR value, suggesting a high anti-tumor activity of effector T cells in tumors of this subtype. Therefore, the barrier for immunotherapy in this subtype seemed more from impaired in immune chemotaxis, immune presentation, and expansion, but which has rare relation with CTNNB1 mutations and PD1/PD-L1. We noted that mHCC3 was characterized by significant altered O-Glycan biosynthesis metabolisms. Although the impact of tumor glycans on anti-tumor immunity has not yet been fully elucidated, the abundant and aberrant cancer glycosylation profile is currently accepted as a distinct hallmark of cancer, and tumor-associated O-glycans bind a variety of receptors on immune cells to facilitate the subsequently induction of immunosuppression (66). Determining whether O-Glycan metabolism could play a dual role as both metabolism and immunotherapy targets requires further investigation.

In summary, our study introduced a novel metabolic class in HCC cases, which comprise not only metabolic heterogeneity but also immune heterogeneity. The metabolic heterogeneity demonstrated that some reprogrammed metabolic pathways affected tumor progression in different rates depending on the subtype, which supports the development of subtype-specific treatment strategies targeting unique metabolic vulnerabilities. The immune heterogeneity across metabolic subtypes suggested that it might be effective to select different immune therapeutic strategies according to different metabolic subtypes. The correlation between immune characters and metabolic features also helped us to find an individualized therapeutic target, the PPT1. Further investigations of the effect of metabolism pathways on both tumor progression and immunologic microenvironment is worthy of study.

## Data availability statement

Publicly available datasets were analyzed in this study. This data can be found here: TCGA database (https://portal.gdc.cancer.gov/), GEO database (https://www.ncbi.nlm.nih.gov/geo/),Molecular Signature Database (MSigDB, https://www.gsea-msigdb.org/gsea/msigdb).The datasets presented in this study can be found in online repositories. The names of the repository/repositories and accession number(s) can be found in the article/**Supplementary Figures and Method**.

## Ethics statement

The animal study was reviewed and approved by the Guangdong Provincial People’s Hospital and the Animal Experimental Research Ethics Committee of South China University of Technology. [Approval number: KY-N-2022-130-01].

## Author contributions

RFC and YZ conceived and designed the study. JY, LZ, and RWC collected and analyzed the data. JY and LZ performed *in vitro* and vivo studies. SZ and RWC review the literature. JY, LZ and RFC wrote the manuscript. All authors contributed to the article and approved the submitted version.
