# Supplementary material for: Characterization of heterogeneous metabolism in hepatocellular carcinoma identifies new therapeutic target and treatment strategy
Source: Front Immunol. 2023 Mar 16;14:1076587. doi: 10.3389/fimmu.2023.1076587 (PMC10060979; doi:10.3389/fimmu.2023.1076587)
Supplement: Supplementary file 1 [file DataSheet_1.pdf]

Supplementary Figures and Method

Characterization of heterogeneous metabolism in hepatocellular carcinoma identifies new therapeutic target and treatment strategy

Jiabin Yang[1,2,3†], Liangtang Zeng[1,2,3†], Ruiwan Chen[4†], Shangyou Zheng[2,3], Yu Zhou[2,3*], Rufu Chen[1,2,3*]

[1] School of Medicine, South China University of Technology, Guangzhou, Guangdong, China.

[2] Department of Pancreatic Surgery, Guangdong Provincial People's Hospital (Guangdong Academy of Medical Sciences), Southern Medical University, Guangzhou , China.

[3] Department of General Surgery, Guangdong Provincial People's Hospital (Guangdong Academy of Medical Sciences), Southern Medical University, Guangzhou , China.

[4] Department of Radiation Oncology, The First Affiliated Hospital, Sun Yat-sen University, Guangzhou, Guangdong, China.

*** Correspondence:**
Rufu Chen, chenrufu@mail.sysu.edu.cn.
Yu Zhou, zhouyu@gdph.org.cn.

†These authors contributed equally to this work and share first authorship

1 Supplementary Figure

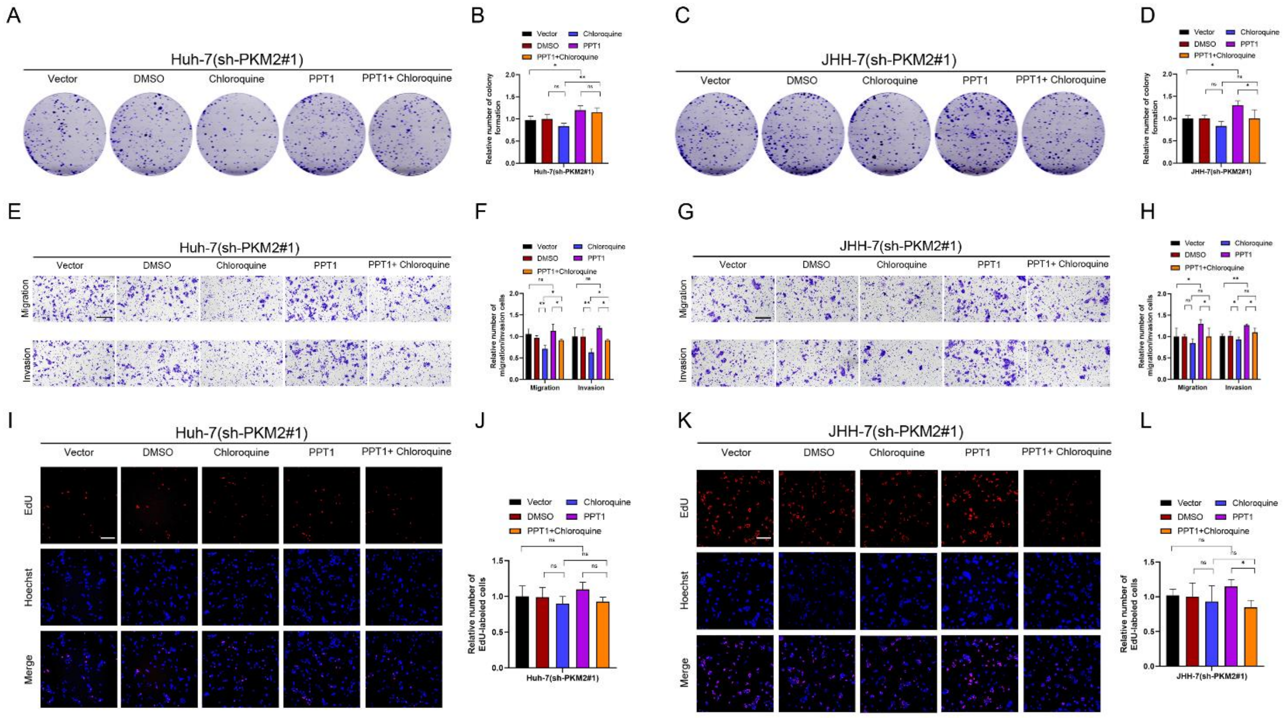

Supplementary Figure S1. The function of PPT1 in stably knockdown PKM2 HCC cells. (A-D) The colony formation was counted in stably knockdown PKM2 between Huh-7 (A, B) and JHH-7 (C, D) cells. (E-H) Representative images and histogram analysis of Transwell migration and Matrigel invasion assays in stably knockdown PKM2 between Huh-7 (E, F) and JHH-7 (G, H) cells. Scale bars: 100 μm. (I-L) Representative images and histogram analysis of EdU assays in stably knockdown PKM2 between Huh-7 (I, J) and JHH-7 (K, L) cells. Scale bars: 100 μm. The error bars are expressed as the mean ± SD of three independent experiments. (ns: not significant, *: $P < 0.05$, **: $P < 0.01$, ***: $P < 0.001$)

Figure 8A

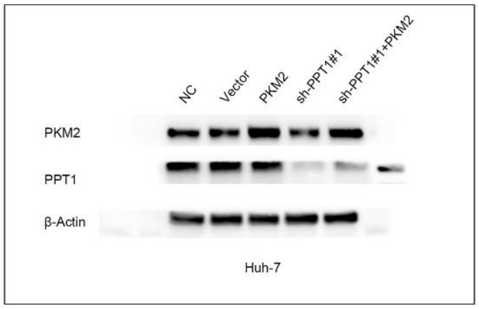

Figure 8B

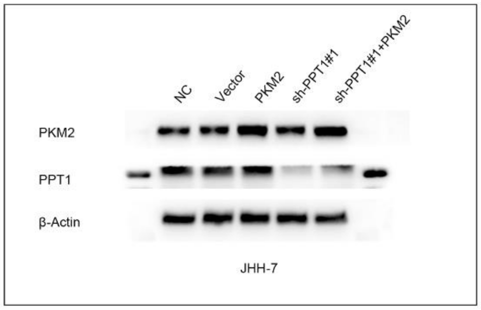

Figure 9M

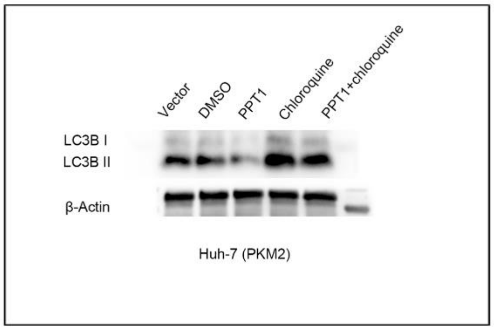

Figure 9N

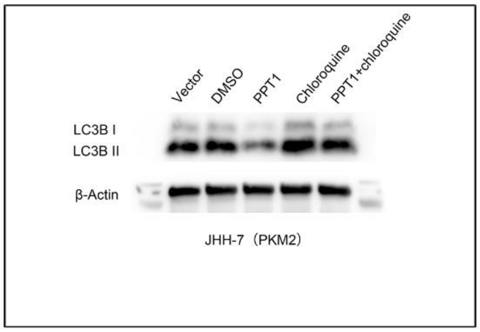

Figure 10A

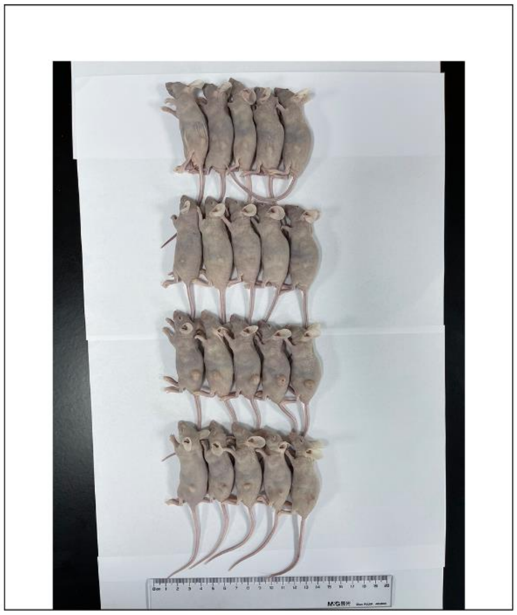

Figure 10B

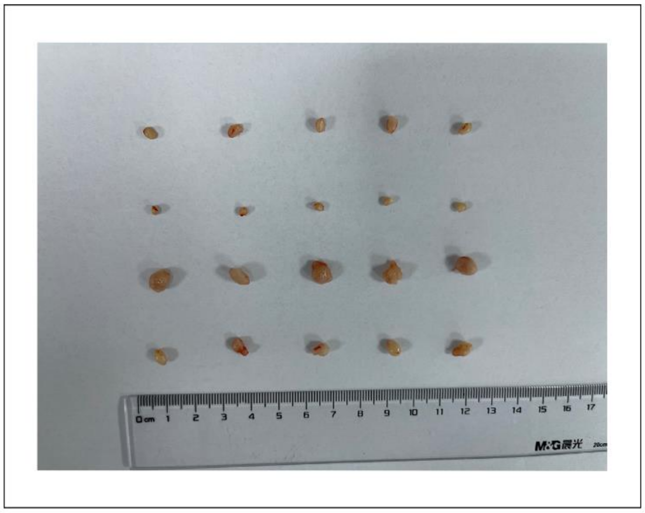

Supplementary Figure S2. Full uncut original pictures.

2 Supplementary Method

Colony formation assay

Five hundred stably overexpressed and knockdown PKM2 Huh-7 and JHH-7 cells were seeded into 6-well plates, treated or transfected with Vector, DMSO, Chloroquine (NSC-187208, Selleckchem, Houston, TX, USA), PPT1 lentivirus and PPT1 lentivirus + Chloroquine, respectively. Cultured in 37 °C containing 5% CO_2 for 2 weeks. The cells were washed three times with phosphate buffered saline (PBS), then fixed with 4% paraformaldehyde for 15 min, and finally stained with 0.1% crystal violet for 15 min. Cell colonies are counted manually. Each group conducted three separate experiments.

5-Ethynyl-20-deoxyuridine (EdU) assay

EdU assays adopted BeyoClickTM EdU-555 detection kits (Beyotime, Shanghai, China) to evaluate HCC cells proliferation. Stably overexpressed and knockdown PKM2 Huh-7 and JHH-7 cells treated or transfected with Vector, DMSO, Chloroquine, PPT1 lentivirus and PPT1 lentivirus + Chloroquine were seeded into 6-well plates for 24 h. The final concentration of 10 μM EdU solution was added to 6-well plates for for 2h. 4% paraformaldehyde was fixed for 15 min. PBS treatment with 0.3% Triton Xmur100 for 15 min. Click reaction solution incubated at room temperature and protected from light for 30 min. 1x Hoechst 33342 solution incubated at room temperature and protected from light for 10 min. All images were photographed by fluorescence microscope Nikon TI-S (Nikon, Tokyo, Japan).

Transwell assay

Transwell assays was used to evaluate the invasion and migration of HCC cells. The cells were seeded in 200 μl serum-free medium that was pre-treated with or without Matrigel (Matrigel BD biosciences, NY, USA) in the upper chamber of 24-well plates, and 700 μl complete medium was added in the lower layer, cultured in 37 °C containing 5% CO_2 for 48h. The upper chamber was fixed with 4% paraformaldehyde for 15 min and stained with 0.1% crystal violet for 15 min. Migrated or invaded cells were captured with light microscope Nikon TI-S (Nikon, Tokyo, Japan) and counted. Each group conducted three separate experiments.

Mouse subcutaneous xenograft models

The BALB/c nude mice aged 4 to 5 weeks were purchased from the Guangdong Medical Laboratory Animal center. A total of 5×10^6 stably overexpressed PKM2 Huh-7 cell suspensions were subcutaneously injected into left hind flank of the mice. On the 7th day after tumor cell transplantation, the mice were randomly divided into four groups (n=5), and injected with Vector (i), Chloroquine (ii), PPT1 lentivirus (iii) and PPT1 lentivirus+ Chloroquine (iv), every 3 days, respectively. The tumor volume was monitored every 4 days, $Volume=0.5 \times length \times width^2$. Four weeks later, all the mice were killed, and the tumor tissue was dissected, measured and weighed. The animal study was reviewed and approved by the Guangdong Provincial People's Hospital and the

Animal Experimental Research Ethics Committee of South China University of Technology. [Approval number: KY-N-2022-130-01].

Immunohistochemistry (IHC)

The paraffin-embedded tissue sections were treated at 60 °C for 2h, then dewaxed with xylene and rehydrated with different gradients of ethanol, the antigens were repaired by EDTA and cooled to room temperature. After being treated with peroxidase blockers, they were sealed in normal goat serum for 30 min and incubated with first antibody at 4 °C overnight. Then, the sections was incubated with secondary antibody at room temperature for 30 min. Finally, the antigen location was marked by avidin-biotin peroxidase detection system based on DAB, and hematoxylin was used for staining. The immunohistochemical signal intensity and positive staining results of tissue sections were independently evaluated and scored by two observers. The antibodies are shown in Supplementary Table S4. All images were captured with microscope Nikon 80i (Nikon, Tokyo, Japan).
